# Successful Treatment of Non-invasive Bladder Cancer During Pregnancy: Diagnostic and Management Challenges

**DOI:** 10.7759/cureus.75417

**Published:** 2024-12-09

**Authors:** Anitha Reddy Depa, Rajitha Reddy Beeravalli, Abhizna Bommiti, Sushama Pawar

**Affiliations:** 1 Obstetrics and Gynecology, Fernandez Hospital, Hyderabad, IND; 2 Radiology, Fernandez Hospital, Hyderabad, IND

**Keywords:** biopsy, bladder cancer, hematuria, pregnancy, turbt

## Abstract

Urological malignancies during pregnancy are exceedingly rare, with bladder cancer posing significant diagnostic and management challenges. This study describes a 28-year-old pregnant woman diagnosed with non-invasive papillary urothelial carcinoma, presenting with painless hematuria at 22 weeks of gestation. The diagnostic process included ultrasound and MRI, both of which confirmed a solitary polypoidal lesion. Surgical management was performed through transurethral resection of bladder tumor (TURBT) under spinal anesthesia at 24 weeks gestation. Challenges included differentiating hematuria from common pregnancy-related conditions and ensuring fetal safety during diagnostic imaging and surgery. The patient delivered a healthy baby at 37 weeks and remains recurrence-free one-year post-surgery. This study underscores the importance of multidisciplinary care and highlights the need for further research into urological cancers in pregnancy to improve outcomes for both mother and fetus.

## Introduction

Malignant tumors during pregnancy occur in approximately 2.35 per 10,000 pregnancies, with bladder cancer being particularly rare, affecting only 13 per 1,000,000 pregnancies. Early diagnosis is critical, as delays can lead to tumor progression and increased maternal and fetal risks. Symptoms such as hematuria are easily misattributed to benign conditions like urinary tract infections, further complicating timely identification. This report illustrates the importance of vigilance and a multidisciplinary approach in managing urological malignancies during pregnancy [1].

## Case presentation

**Figure 1:**
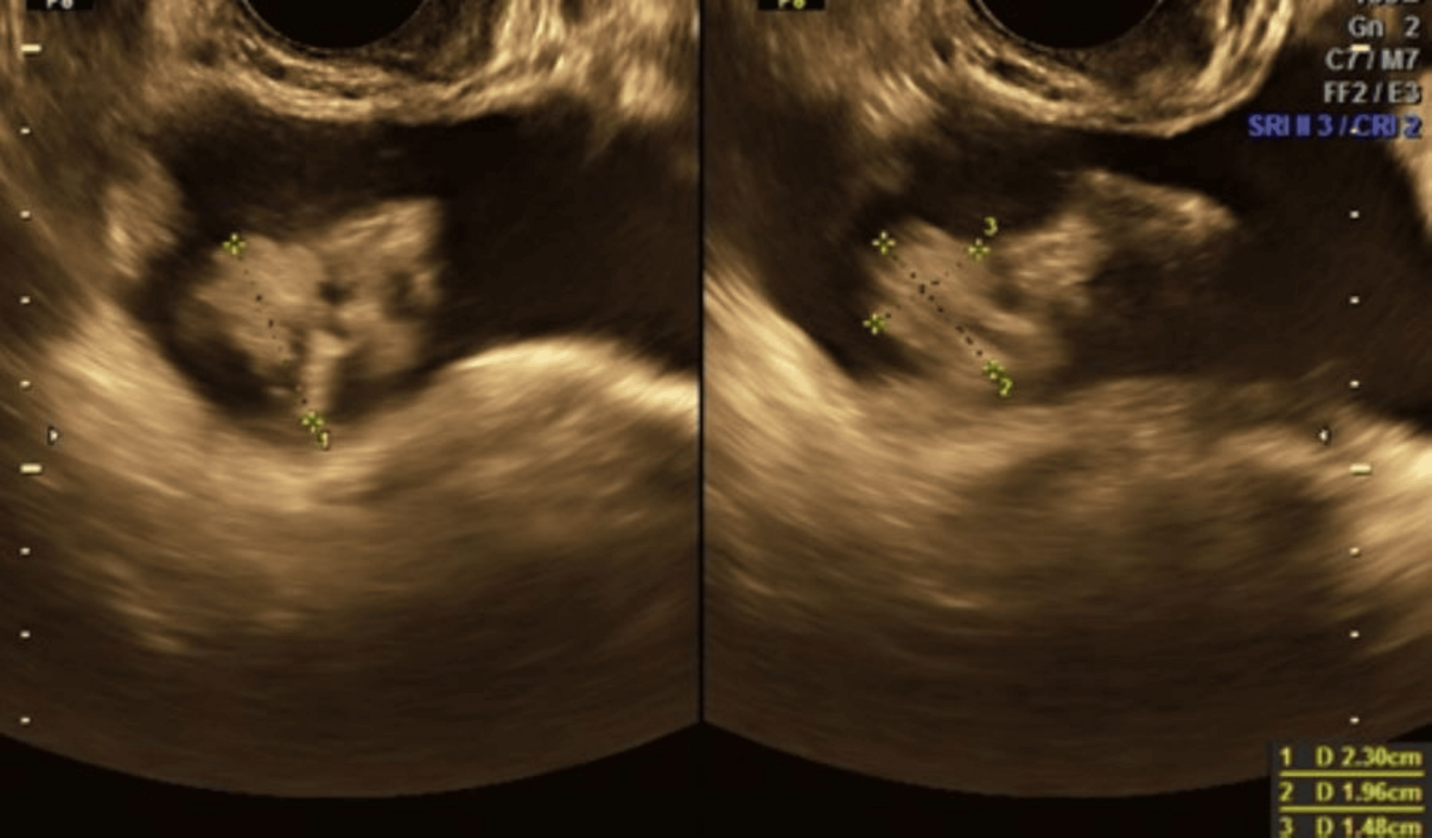
Ultrasonography showing a small echogenic polypoidal lesion measuring 27 × 17 mm along the anterolateral wall of the right side of the urinary bladder.

A 28-year-old woman was registered for care at six weeks gestation with a BMI of 23 kg/m^2^. Her booking antenatal investigations were normal. At 22 weeks, she presented to the emergency room with painless hematuria. She was evaluated with routine urine examination and urine for culture and sensitivity which were normal. Ultrasound examination of the genitourinary system showed normal sono morphology of both kidneys with mild pelvi-ureteric dilatation on the right side. A small polypoidal lesion along the urinary bladder with vascular stalk was seen and a diagnosis of bladder papilloma was made (Figure 1).

She was referred to a urologist for further management. MRI showed a solitary polypoidal T2 isointense lesion with papillary surfaces arising from the right anterior wall of the urinary bladder and a small stalk with no extension of the lesion beyond the bladder wall confines. Imaging findings were in favor of bladder papilloma. Urine cytology was negative for atypical or malignant cells. The patient was investigated thoroughly and after preoperative evaluation and preanesthetic checkup, surgical management was planned. At 24 weeks of gestation, transurethral resection of bladder tumor (TURBT) was done.

Surgical procedure

**Figure 2:**
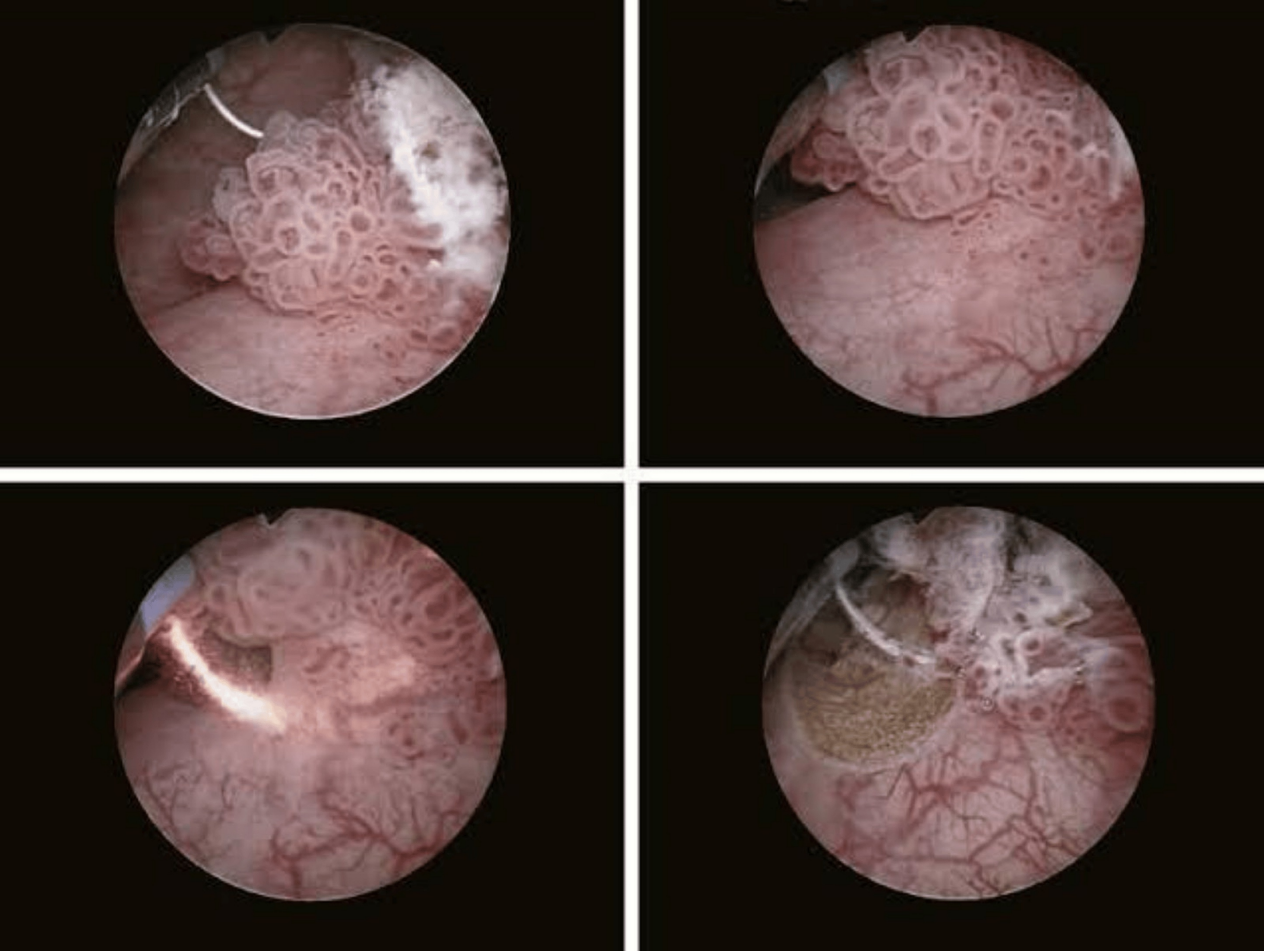
Cystoscopy and transurethral resection of bladder tumor (TURBT). TURBT: transurethral resection of bladder tumor

Cystoscopy showed a solitary papillary lesion in the dome of the bladder. Urethra and ureteric orifices were normal. Transurethral resection of the bladder tumor was done under spinal anesthesia (Figure 2). Superficial and deep muscle biopsy was taken and sent for histopathological examination (HPE). A 20 Fr three-way urethral catheter was placed. The intra and postoperative period was uneventful. The urethral catheter was removed on the second postoperative day. During the hospital stay the patient was treated with appropriate antibiotics and analgesics. HPE showed low-grade non-invasive papillary urothelial carcinoma.

Follow-up antenatal care

Thereafter, she had regular antenatal visits. She was detected to have gestational diabetes at 28 weeks, which was managed by medical nutritional therapy.

Fetus

Her prenatal screening ultrasound at 13 weeks showed a low risk for trisomy 21, 13, and 18 and screened negative for preeclampsia. Targeted imaging for fetal anomalies scan (TIFFA) was done at 20 weeks and showed no obvious detectable anomalies. Fetal growth surveillance was done by ultrasound examination from 28 weeks onwards.

Labor and birth

At 37 weeks, induction of labor was done for fetal growth restriction and she had a successful vaginal birth, birthed a healthy baby boy weighing 2.64 kg with good Apgar and normal cord gases. The mother and baby were discharged on the first postnatal day in stable condition.

She had a regular postnatal visit at six weeks with no complaints. She is continuing follow-ups with a urologist. Cystoscopy after six months of surgery was normal. One year post-surgery, the patient remains in good health with no evidence of recurrence.

## Discussion

Bladder cancer during pregnancy is an exceptionally rare and complex medical condition, posing significant challenges for both diagnosis and treatment. It requires a coordinated, multidisciplinary approach involving experts from urology, oncology, obstetrics, and psychology to ensure the best outcomes for both mother and fetus [2].

The initial symptoms of bladder cancer, particularly hematuria, are easily confused with common pregnancy-related changes, such as increased urinary tract infections (UTIs) or other urological issues. This can delay accurate diagnosis, especially when hematuria occurs without accompanying pain, or when recurrent bladder infections (as seen in several reported cases) mislead the clinician [3].

For diagnosis, cystoscopy remains the gold standard but poses challenges due to its invasive nature, especially during pregnancy. Non-invasive options such as ultrasound are safer for the fetus but often provide limited clarity when it comes to identifying bladder malignancies. MRI is another safer imaging alternative during pregnancy, as it avoids radiation exposure, but it has limitations in distinguishing the individual layers of the bladder wall, reducing its diagnostic accuracy. CT scans, though highly effective, are typically avoided because of fetal concerns [3,4].

Given these constraints, clinicians may take a more conservative approach, relying heavily on non-invasive methods such as ultrasound, despite their limitations. While MRI can be a safer option, it doesn’t fully overcome the diagnostic challenges, particularly the inability to perform a biopsy without cystoscopy [5].

Treating bladder cancer during pregnancy demands careful balancing between effective treatment for the mother and the safety of the fetus. The factors, which decide the treatment are the trimester of pregnancy at which the patient has presented and the stage of malignancy [6]. Standard treatments, such as surgery or chemotherapy, may require adjustments, as certain drugs and anesthetic agents pose risks to the developing fetus. The use of anesthesia during procedures must be considered with great caution to minimize fetal risk while prioritizing the mother's health [7]. Specialized anesthesia considerations were crucial in the present case to ensure maternal and fetal safety during surgery. Spinal anesthesia was chosen to minimize systemic drug exposure and reduce risks associated with general anesthesia, such as uteroplacental perfusion compromise. The anesthetic team closely monitored maternal hemodynamics and fetal well-being throughout the procedure, ensuring a stable intraoperative environment. This approach highlights the critical role of anesthesiology in balancing effective surgical management with fetal safety. In this case of non-invasive papillary urothelial carcinoma, the standard treatment remains transurethral resection of bladder tumor (TURBT), as it is similar to the management in non-pregnant individuals.

Beyond the medical complexities, there is a substantial psychological toll on the pregnant woman diagnosed with bladder cancer. The emotional strain of dealing with cancer, coupled with the fear of affecting the unborn child, can be overwhelming. This adds an extra layer of stress, as the patient must weigh difficult decisions about treatment while managing anxiety over the baby’s well-being. Psychological support, therefore, becomes a critical component of care, ensuring that the patient and her family are supported throughout this challenging journey [8].

The epidemiology of bladder cancer during pregnancy is not well understood, as it is extremely rare, with few documented cases. This scarcity of data highlights the need for further research, particularly through prospective studies, to better understand the incidence and prevalence of this condition. Such studies would also contribute to developing more evidence-based guidelines for diagnosing and managing bladder cancer in pregnant women.

## Conclusions

This case highlights the importance of early detection and evaluation of hematuria in pregnancy, emphasizing the role of imaging modalities like ultrasound and MRI in diagnosis while minimizing fetal risks. Bladder cancer during pregnancy remains an under-researched area, necessitating further studies to establish evidence-based guidelines for diagnosis, anesthesia management, and treatment. Prospective research on the epidemiology, diagnostic pathways, and multidisciplinary approaches will be critical in improving outcomes for this rare but significant condition.
